# Comparison of clinical and radiological outcomes for the anterior and medial approaches to open reduction in the treatment of bilateral developmental dysplasia of the hip: a systematic review protocol

**DOI:** 10.1186/s13643-023-02444-6

**Published:** 2024-02-23

**Authors:** Edward Alan Jenner, Govind Singh Chauhan, Abdus Burahee, Junaid Choudri, Adrian Gardner, Christopher Edward Bache

**Affiliations:** 1 Birmingham Children’s Hospital, Steelhouse Lane, Birmingham, B4 6NH UK; 2 Royal Orthopaedic Hospital, Bristol Road South, Birmingham, B31 2AP UK; 3 University of Birmingham, College of Medical & Dental Sciences, Birmingham, UK

**Keywords:** Developmental dysplasia of the hip, Congenital dysplasia of the hip, Congenital hip dislocation, Developmental hip dislocation, DDH, CDH, Bilateral

## Abstract

**Background:**

Developmental dysplasia of the hip (DDH) affects 1–3% of newborns and 20% of cases are bilateral. The optimal surgical management strategy for patients with bilateral DDH who fail bracing, closed reduction or present too late for these methods to be used is unclear. There are proponents of both medial approach open reduction (MAOR) and anterior approach open reduction (AOR); however, there is little evidence to inform this debate.

**Methods:**

We will perform a systematic review designed according to the Preferred Reporting Items for Systematic Review and Meta-Analysis Protocol. We will search the medical and scientific databases including the grey and difficult to locate literature. The Medical Subject Headings “developmental dysplasia of the hip”, “congenital dysplasia of the hip”, “congenital hip dislocation”, “developmental hip dislocation”, and their abbreviations, “DDH” and “CDH” will be used, along with the qualifier “bilateral”. Reviewers will independently screen records for inclusion and then independently extract data on study design, population characteristics, details of operative intervention and outcomes from the selected records. Data will be synthesised and a meta-analysis performed if possible. If not possible we will analyse data according to Systematic Review without Meta-Analysis guidance. All studies will be assessed for risk of bias. For each outcome measure a summary of findings will be presented in a table with the overall quality of the recommendation assessed using the Grading of Recommendations Assessment Development and Evaluation approach.

**Discussion:**

The decision to perform MAOR or AOR in patients with bilateral DDH who have failed conservative management is not well informed by the current literature. High-quality, comparative studies are exceptionally challenging to perform for this patient population and likely to be extremely uncommon. A systematic review provides the best opportunity to deliver the highest possible quality of evidence for bilateral DDH surgical management.

**Systematic review registration:**

The protocol has been registered in the International Prospective Register of Systematic Reviews (PROSPERO ID CRD42022362325).

**Supplementary Information:**

The online version contains supplementary material available at 10.1186/s13643-023-02444-6.

## Introduction

DDH describes a spectrum of abnormalities in the infant’s hip, from subluxation to frank dislocation, due to incomplete acetabular and femoral head development [1]. Developmental dysplasia of the hip (DDH) affects 1–3% of newborns and 20% of cases are bilateral [2–4]. Although many cases of DDH spontaneously resolve as the child grows [5] those in whom the hip(s) remains shallow, subluxed, or dislocated will go on to develop gait abnormalities, hip pain, and early onset osteoarthritis [6]. This often requires early hip arthroplasty [7]. Clinical and radiological outcomes for children with bilateral DDH have been reported to be worse than for children with unilateral DDH by some authors [8–10] whereas others have found no difference [11, 12].

The aim of treatment in bilateral DDH is to achieve concentrically reduced hips, without significant deformity or residual dysplasia. If bilateral DDH is detected as a neonate, abduction bracing is attempted, although failure rates are higher than for unilateral disease [8, 13, 14]. Patients who fail bracing proceed to examination under anesthetic and arthrogram, aiming for closed reduction and hip spica. Typically, this is performed before age 6 months. Bilateral DDH represents a significant risk factor for failure of conservative treatment [8, 9] and patients failing closed reduction proceed to open reduction.

Operative options are medial approach open reduction (MAOR) or anterior approach open reduction (AOR). MAOR is performed between 6 and 18 months of age [15]. This approach requires limited soft tissue dissection through a small, cosmetically acceptable, anteromedial incision with minimal blood loss. The anatomical blocks to reduction (capsular constriction, transverse acetabular ligament, ligamentum teres and iliopsoas tendon) are well visualised and released. Both hips are usually operated on at the same sitting and the patient is immobilised in a hip spica for 6–12 weeks postoperatively. Critics suggest that MAOR increases the risk of femoral head avascular necrosis (AVN), prevents the blocks to femoral head reduction from being fully addressed and does not allow capsulorrhaphy [16–18]. Rates of residual dysplasia may also be higher. It has been reported that MAOR may have worse outcomes compared to AOR [16–18]; however, these studies relate to unilateral cases and limited data, specific to bilateral DDH, has been published. The data relating to unilateral disease is itself heterogeneous and contradictory [15, 19, 20].

AOR is usually performed around 12–24 months of age through a bikini line incision via the ilio-inguinal approach. This results in a larger, less cosmetically acceptable scar, more soft tissue dissection, potentially greater blood loss and risks of damage to the lateral femoral cutaneous nerve [21, 22]. Proponents argue that AOR allows all the potential blocks to femoral head reduction to be addressed and capsulorrhaphy to be performed therefore improving outcomes [23]. Pelvic osteotomy can be performed through the same approach and this is usually required when surgery is undertaken after age 2 years [24–27]. Typically, in AOR, one hip is operated on at each sitting with a 6-week gap between surgeries during which the patient is immobilised in a hip spica cast [11, 12]. Some authors have reported single-sitting bilateral surgery in AOR [25]; however, this remains rare.

The choice of AOR or MAOR depends on a number of factors, including the patient’s age, the surgeon’s training and experience and the perceived advantages and disadvantages of each technique. Both of these surgical management strategies for bilateral DDH have proponents on each side, however, there is limited evidence to inform decision-making. To the best of our knowledge, this will be the first systematic review comparing outcomes for AOR vs MAOR in bilateral DDH.

## Aims

Our aim is to establish whether there is a difference in the clinical and radiological outcomes for children with bilateral DDH who have been treated with MAOR compared to AOR. We will examine a range of clinical and radiological outcome measures and if possible perform a quantitative analysis. We will summarise the evidence available and give recommendations for management. This will help to inform decision-making in the management of bilateral DDH.

## Design and methods

This protocol has been designed according to the Preferred Reporting Items for Systematic Review and Meta-Analysis Protocol (PRISMA-P) [28, 29]. The design and method have been formed through discussion between experts in the management of DDH and experts in the methodology of systematic reviews. The protocol has been registered in the International Prospective Register of Systematic Reviews (PROSPERO ID-CRD42022362325).

### Eligibility criteria

#### Population

Children with idiopathic bilateral developmental dysplasia of the hip undergoing surgical management of both hips.

Exclusion criteria—children with bilateral DDH in whom one hip is managed through harness treatment alone, children with teratologic bilateral developmental dysplasia of the hip, children undergoing revision surgery and surgery for acetabular dysplasia in adolescence.

#### Intervention

Medial approach open reduction of the hip (MAOR).

#### Comparison

Anterior approach open reduction of the hip (AOR).

#### Outcomes

1. Rate and severity of avascular necrosis of the femoral head at the latest follow-up using Kalamchi and MacEwen [30] or Bucholz and Ogden classification [31] or other appropriate scoring system.
*and/or*
2. Radiological outcome at the latest follow-up using acetabular index measured in degrees, Severin Score [32] or other appropriate scoring system.
*and/or*
3. Clinical outcomes at the latest follow-up including Modified McKay criteria [33], Children’s Hospital of Oakland Hip Evaluation Scale [34], Pediatric Outcomes Data Collection Instrument (PODCI) [35] or other appropriate scoring system.
*and/or*
4. Prevalence, event rate or time-to-event surgical complications assessed according to the Clavien-Dindo system [36, 37] or other appropriate scoring system.
*and/or*
5. Prevalence, event rate or time to event of secondary surgery.

#### Study design

Inclusion criteria—clinical studies, level IV (retrospective case series) and above, with a clear description of the operative management with a set of clinical and/or radiological outcomes included, published in English.

Exclusion criteria—case reports, technical or cadaveric studies, studies without a clear description of the operative management or where this is unobtainable, studies without a clear description of clinical and/or radiological outcomes or where this is unobtainable. Full-text studies not available in English will be excluded.

### Search strategy

A search of the electronic medical and scientific databases; PubMed, MEDLINE, the Cochrane Library, Embase, Google Scholar, Web of Science and Scopus will be conducted from the date of first entry until the date of search. The grey and difficult-to-locate literature (including theses and dissertations) will be searched via the Open Grey [38] and Open Access Theses and Dissertations [39] databases. The Medical Subject Headings (MeSH terms) “developmental dysplasia of the hip”, “congenital dysplasia of the hip”, “congenital hip dislocation”, “developmental hip dislocation”, and their abbreviations, “DDH” and “CDH” will be used, along with the qualifier “bilateral”. The search strategy will be developed in Medline and then applied to other databases. An example of the search strategy can be found in Additional file 1. Only full-text studies, published in English will be included. There will be no time limit imposed.

### Study selection

Two reviewers (EJ and GC) will independently screen the title and abstract of records for inclusion according to the eligibility criteria. Once preliminary screening has been performed, selected studies will be screened as full text. Researchers will be blinded to each other’s decisions. Where there is disagreement a separate reviewer (CEB) will arbitrate. Screening decisions at the full-text stage will be fully recorded. The results of the screening will be presented in a PRISMA flow diagram [29].

### Data management

The selected studies will be collated in the Zotero citation management system, screened for duplicates, and exported to Systematic Review Data Repository-Plus [40]. This database will be used to aid data extraction and management. Extracted data will be exported to RevMan software for analysis.

### Data extraction

Data will be extracted in a predefined electronic data extraction form. Data on study design, population characteristics, details of operative intervention (intervention and comparison), and outcomes (clinical, radiological, complications and rate of secondary surgery) will be extracted. A summary of intended data items for extraction is shown in Table 1. Four reviewers (EJ, GC, MJC and AB) will be allocated the selected studies and will independently extract data. Each reviewer will be blinded to data extraction. Where possible corresponding authors will be contacted for unreported data. Data will be extracted to a secured anonymised form on Systematic Review Data Repository-Plus and then exported to RevMan for analysis.

**Table 1:** Items included for data extraction in selected studies

Data item	Details
Study details	Reference, year of publication, geographical location of study, study design, ethical approval, funding, pre-registered protocol
Population details	Number of patients, age, sex, comorbidities, inclusion/exclusion critieria, duration of follow-up, DDH severity or classification, presence of ossific nucleus
Surgical intervention	Anaesthetic used (incl. Nerve blocks/ spinal), single stage or sequential surgery, Surgical approach, anatomical details of the approach, blocks to reduction addressed and how addressed, presence or absence of ligamentum teres, surgical duration, the volume of blood loss, details of additional procedures (e.g. pelvic osteotomy/femoral osteotomy), method of post-operative immobilisation, duration of post-operative immobilisation
Clinical outcomes including hip-specific outcome scores and general quality of life scores	E.g. Modified McKay criteria [33], Children’s Hospital of Oakland Hip Evaluation Scale [34], Pediatric Outcomes Data Collection Instrument (PODCI) [35]
Radiological outcomes including hip and age-specific outcome scores	E.g. rate and severity of AVN (Kalamchi and MacEwen [30] or Bucholz and Ogden classification [31]), acetabular index measured in degrees, Severin Score [32]
Surgical outcomes	Surgical compilation rate, surgical complications according to Clavien-Dindo classification, dislocation rate, secondary surgery

### Data synthesis

The extracted data will be summarised in a structured table format, grouped and ordered by study design (according to the hierarchy of evidence) or by risk bias if study designs are similar, and including the data items specific to the outcomes of interest. This will help to assess clinical and methodological heterogeneity across the studies and determine the feasibility of performing a meta-analysis. We do not expect the included studies to be of sufficient quality or consistency to allow a meta-analysis to be performed. In this instance, we will follow the Systematic Review without Meta-Analysis (SWiM) guidance [41] and analyse data according to this and the recommendations in the Cochrane Handbook Chapter 12 [42]. Studies will be grouped and tabulated as described. We expect that the key outcome data for radiological and clinical outcomes will be in short ordinal scales (e.g. Severin Score [32]). Where possible we will transform these data to dichotomous outcomes and present this as a relative risk with 95% confidence intervals for MAOR in comparison to AOR. Longer ordinal scales such as the Pediatric Outcomes Data Collection Instrument [35] will be transformed to continuous data. For complications and secondary surgery data we will transform to an incidence estimate, event rate or time-to-event data. For non-comparative studies, we will transform extracted data as described above and use this to generate a crude estimate of incidence, prevalence or event rate. Where possible we will pool this data using a random effects model as per the recommendation in Murad et al. [43]. Results will be reported according to the guidance in the Cochrane Handbook Chapter 12 [42]. Where sufficient information is available but synthesis cannot be performed a structured reporting of effects will be used. When effect estimates are available without measures of precision an illustrated synthesis of summary statistics will be used. If *P* values are available an illustrated synthesis of *P* values will be used. Where directions of effect are available an illustrated synthesis using vote-counting based on direction of effect will be used.

### Meta-bias

We aim to limit publication bias by a thorough and systematic search of the literature including the grey literature as described in the search strategy. Where possible publication bias will be assessed across studies by generation of funnel plots. These will be inspected for asymmetry and analysed via Egger’s test [44].

### Risk of bias

Randomised trials will be assessed using the Cochrane Risk of Bias 2 (RoB 2) tool [45]. However, included studies are most likely to be non-randomised, observational studies. For comparative studies (cohort or case–control) we will use the ROBINS-I tool to assess risk of bias [46]. For case series, we will use Murad et al.*’s* method for evaluating the methodological quality across four domains; selection, ascertainment, causality and reporting [43]. Four reviewers (EJ, GC, MJC & AB) will assess included studies for risk of bias. A separate reviewer (CEB) will resolve disagreements through discussion. A summary figure of the risk of bias analysis will be included in the final manuscript.

### Assessment of quality

For each outcome measure a summary of findings will be presented in a table [47] with the overall quality of the recommendation assessed using the Grading of Recommendations Assessment Development and Evaluation approach (GRADE) [48]. This approach uses five factors; risk of bias, inconsistency, indirectness, imprecision and publication bias to assess the quality of evidence and produce a rating of “high”, “moderate”, “low” or “very low”. GRADEpro GDT software [49] will be used to aid decision-making when assessing the quality of evidence.

### Discussion and implications of review

Management of bilateral DDH represents a significant challenge for the paediatric orthopaedic surgeon. The aim of treatment is to achieve concentrically reduced hips, without significant deformity or residual dysplasia. The decision to perform MAOR or AOR in patients with bilateral DDH who have failed conservative management is not well informed by the current literature. High-quality, comparative studies are exceptionally challenging to perform for this patient population and likely to be extremely uncommon. A systematic review provides the best opportunity to deliver the highest possible quality of evidence for bilateral DDH surgical management. We are not aware of any systematic reviews that compare the outcomes of MAOR with AOR for bilateral DDH. This study aims to identify whether there are any significant differences in the clinical or radiological outcomes for patients with bilateral DDH surgically treated with MAOR compared to AOR so that surgeons can make better-informed decisions about the management strategy they will offer to patients.

### Limitations

We expect that this review will be limited by studies that have a small sample size and have a retrospective, non-comparative study design. We expect result reporting to be heterogeneous and incomplete. These limitations will place all studies at a high risk of bias and therefore limit the quality of evidence that can be derived from the systematic review.

### Supplementary Information

**Additional file 1.** Search strategy example.

## Data Availability

Not applicable.

## Abbreviations

DDH: Developmental dysplasia of the hip
MAOR: Medial approach open reduction
AOR: Anterior approach open reduction
CDH: Congenital dysplasia of the hip
AVN: Avascular necrosis
PRISMA-P: Preferred Reporting Items for Systematic Review and Meta-Analysis Protocol
PODCI: Pediatric Outcomes Data Collection Instrument
MeSH: Medical Subject Headings
SWiM: Systematic Review without Meta-Analysis
RoB 2: Cochrane Risk of Bias 2
GRADE: Grading of Recommendations Assessment Development and Evaluation approach

## References

[1] Zhang S, Doudoulakis KJ, Khurwal A, Sarraf KM (2020). Developmental dysplasia of the hip. Br J Hosp Med Lond Engl 2005.

[2] Sewell MD, Rosendahl K, Eastwood DM (2009). Developmental dysplasia of the hip. BMJ.

[3] Marks DS, Clegg J, Al-Chalabi AN (1994). Routine ultrasound screening for neonatal hip instability. Can it abolish late-presenting congenital dislocation of the hip?. J Bone Joint Surg Br.

[4] Macnicol MF (1990). Results of a 25-year screening programme for neonatal hip instability. J Bone Joint Surg Br.

[5] Bialik V, Bialik GM, Blazer S, Sujov P, Wiener F, Berant M (1999). Developmental dysplasia of the hip: a new approach to incidence. Pediatrics.

[6] Cooperman DR, Wallensten R, Stulberg SD (1983). Acetabular dysplasia in the adult. Clin Orthop.

[7] Furnes O, Lie SA, Espehaug B, Vollset SE, Engesaeter LB, Havelin LI (2001). Hip disease and the prognosis of total hip replacements. A review of 53,698 primary total hip replacements reported to the Norwegian Arthroplasty Register 1987–99. J Bone Joint Surg Br.

[8] Kitoh H, Kawasumi M, Ishiguro N (2009). Predictive factors for unsuccessful treatment of developmental dysplasia of the hip by the Pavlik harness. J Pediatr Orthop.

[9] Viere RG, Birch JG, Herring JA, Roach JW, Johnston CE (1990). Use of the Pavlik harness in congenital dislocation of the hip. An analysis of failures of treatment. J Bone Joint Surg Am.

[10] Greene WB, Drennan JC (1982). A comparative study of bilateral versus unilateral congenital dislocation of the hip. Clin Orthop.

[11] Zionts LE, MacEwen GD (1986). Treatment of congenital dislocation of the hip in children between the ages of one and three years. J Bone Joint Surg Am.

[12] Wang TM, Wu KW, Shih SF, Huang SC, Kuo KN (2013). Outcomes of open reduction for developmental dysplasia of the hip: does bilateral dysplasia have a poorer outcome?. J Bone Jt Surg Am.

[13] Segal LS, Boal DK, Borthwick L, Clark MW, Localio AR, Schwentker EP (1999). Avascular necrosis after treatment of DDH: the protective influence of the ossific nucleus. J Pediatr Orthop.

[14] Lerman JA, Emans JB, Millis MB, Share J, Zurakowski D, Kasser JR (2001). Early failure of Pavlik harness treatment for developmental hip dysplasia: clinical and ultrasound predictors. J Pediatr Orthop.

[15] Akilapa O (2014). The medial approach open reduction for developmental dysplasia of the hip: do the long-term outcomes validate this approach? A systematic review of the literature. J Child Orthop.

[16] Okano K, Yamada K, Takahashi K, Enomoto H, Osaki M, Shindo H (2009). Long-term outcome of Ludloff’s medial approach for open reduction of developmental dislocation of the hip in relation to the age at operation. Int Orthop.

[17] Mankey MG, Arntz GT, Staheli LT (1993). Open reduction through a medial approach for congenital dislocation of the hip. A critical review of the Ludloff approach in sixty-six hips. J Bone Joint Surg Am.

[18] Koizumi W, Moriya H, Tsuchiya K, Takeuchi T, Kamegaya M, Akita T (1996). Ludloff’s medial approach for open reduction of congenital dislocation of the hip. A 20-year follow-up. J Bone Joint Surg Br.

[19] Gardner ROE, Bradley CS, Howard A, Narayanan UG, Wedge JH, Kelley SP (2014). The incidence of avascular necrosis and the radiographic outcome following medial open reduction in children with developmental dysplasia of the hip: a systematic review. Bone Jt J.

[20] Hoellwarth JS, Kim YJ, Millis MB, Kasser JR, Zurakowski D, Matheney TH (2015). Medial versus anterior open reduction for developmental hip dislocation in age-matched patients. J Pediatr Orthop.

[21] Jia G, Wang E, Lian P, Liu T, Zhao S, Zhao Q (2020). Anterior approach with mini-bikini incision in open reduction in infants with developmental dysplasia of the hip. J Orthop Surg.

[22] Rudin D, Manestar M, Ullrich O, Erhardt J, Grob K (2016). The anatomical course of the lateral femoral cutaneous nerve with special attention to the anterior approach to the hip joint. JBJS.

[23] Herring JA Tachdjian MO. Texas Scottish rite hospital for children. Tachdjian’s Pediatric Orthopaedics. 4th ed. Philadelphia: Saunders/Elsevier; 2008. help_tachdjiansv1c15.pdf. Available from: https://storage.googleapis.com/global-help-publications/books/help_tachdjiansv1c15.pdf. [cited 2023 Sep 25].

[24] Subasi M, Arslan H, Cebesoy O, Buyukbebeci O, Kapukaya A (2008). Outcome in unilateral or bilateral DDH treated with one-stage combined procedure. Clin Orthop.

[25] Ezirmik N, Yildiz K (2012). Advantages of single-stage surgical treatment with salter innominate osteotomy and pemberton pericapsular osteotomy for developmental dysplasia of both hips. J Int Med Res.

[26] Agus H, Bozoglan M, Kalenderer Ö, Kazımoğlu C, Onvural B, Akan İ (2014). How are outcomes affected by performing a one-stage combined procedure simultaneously in bilateral developmental hip dysplasia?. Int Orthop.

[27] Kotzias Neto A, Ferraz A, Bayer Foresti F, Barreiros HR (2014). Bilateral developmental dysplasia of the hip treated with open reduction and Salter osteotomy: analysis on the radiographic results. Rev Bras Ortop Engl Ed.

[28] Rethlefsen ML, Kirtley S, Waffenschmidt S, Ayala AP, Moher D, Page MJ (2021). PRISMA-S: an extension to the PRISMA statement for reporting literature searches in systematic reviews. Syst Rev.

[29] Moher D, Shamseer L, Clarke M, Ghersi D, Liberati A, Petticrew M (2015). Preferred reporting items for systematic review and meta-analysis protocols (PRISMA-P) 2015 statement. Syst Rev.

[30] Kalamchi A, MacEwen GD (1980). Avascular necrosis following treatment of congenital dislocation of the hip. J Bone Joint Surg Am.

[31] Patterns of ischemic necrosis of the proximal femur in nonoperatively treated congenital hip disease – ScienceOpen. Available from: https://www.scienceopen.com/document?vid=397821b3-1187-46ae-a255-9f9805906ecf. [cited 2022 Oct 12].

[32] Severin E (1950). Congenital dislocation of the hip; development of the joint after closed reduction. J Bone Joint Surg Am.

[33] McKay DW (1974). A comparison of the innominate and the pericapsular osteotomy in the treatment of congenital dislocation of the hip. Clin Orthop.

[34] Aguilar CM, Neumayr LD, Eggleston BE, Earles AN, Robertson SM, Jergesen HE (2005). Clinical evaluation of avascular necrosis in patients with sickle cell disease: children’s hospital Oakland hip evaluation scale–a modification of the harris hip score. Arch Phys Med Rehabil.

[35] Daltroy LH, Liang MH, Fossel AH, Goldberg MJ (1998). The POSNA pediatric musculoskeletal functional health questionnaire: report on reliability, validity, and sensitivity to change. Pediatric Outcomes Instrument Development Group. Pediatric Orthopaedic Society of North America. J Pediatr Orthop.

[36] Dindo D, Demartines N, Clavien PA (2004). Classification of surgical complications: a new proposal with evaluation in a cohort of 6336 patients and results of a survey. Ann Surg.

[37] Dodwell ER, Pathy R, Widmann RF, Green DW, Scher DM, Blanco JS (2018). Reliability of the modified Clavien-Dindo-Sink complication classification system in pediatric orthopaedic surgery. JBJS Open Access.

[38] OPENGREY.EU - Grey literature database. Available from: https://opengrey.eu/. [cited 2022 Oct 13].

[39] OATD – Open access theses and dissertations. Available from: https://oatd.org/. [cited 2022 Oct 13].

[40] SRDR+. Available from: https://srdrplus.ahrq.gov/. [cited 2022 Oct 12].

[41] Campbell M, McKenzie JE, Sowden A, Katikireddi SV, Brennan SE, Ellis S (2020). Synthesis without meta-analysis (SWiM) in systematic reviews: reporting guideline. BMJ.

[42] Chapter 12: synthesizing and presenting findings using other methods. Available from: https://training.cochrane.org/handbook/current/chapter-12. [cited 2022 Oct 12].

[43] Murad MH, Sultan S, Haffar S, Bazerbachi F (2018). Methodological quality and synthesis of case series and case reports. BMJ Evid Based Med.

[44] Egger M, Davey Smith G, Schneider M, Minder C (1997). Bias in meta-analysis detected by a simple, graphical test. BMJ.

[45] Sterne JAC, Savović J, Page MJ, Elbers RG, Blencowe NS, Boutron I (2019). RoB 2: a revised tool for assessing risk of bias in randomised trials. BMJ.

[46] Sterne JA, Hernán MA, Reeves BC, Savović J, Berkman ND, Viswanathan M (2016). ROBINS-I: a tool for assessing risk of bias in non-randomised studies of interventions. BMJ.

[47] Guyatt G, Oxman AD, Akl EA, Kunz R, Vist G, Brozek J (2011). GRADE guidelines: 1. Introduction-GRADE evidence profiles and summary of findings tables. J Clin Epidemiol.

[48] Guyatt GH, Oxman AD, Vist GE, Kunz R, Falck-Ytter Y, Alonso-Coello P (2008). GRADE: an emerging consensus on rating quality of evidence and strength of recommendations. BMJ.

[49] Guideline Development Tool. Available from: https://gdt.gradepro.org/app/#projects. [cited 2022 Oct 13].

