# Supplementary material for: Comparison of clinical and radiological outcomes for the anterior and medial approaches to open reduction in the treatment of bilateral developmental dysplasia of the hip: a systematic review protocol
Source: Syst Rev. 2024 Feb 23;13:72. doi: 10.1186/s13643-023-02444-6 (PMC10885537; doi:10.1186/s13643-023-02444-6)
Supplement: Supplementary file 1 — Additional file 1. Search strategy example. [file 13643_2023_2444_MOESM1_ESM.pdf]

"Hip Dislocation, Congenital"[Mesh] OR "Hip Dysplasia, Congenital"[Mesh] OR "Hip Dislocation, Developmental"[Mesh] OR "Hip Dysplasia, Developmental"[Mesh] OR "DDH" OR "CDH") AND "Bilateral"[Mesh]
